# Dexamethasone Inhibits Spheroid Formation of Thyroid Cancer Cells Exposed to Simulated Microgravity

**DOI:** 10.3390/cells9020367

**Published:** 2020-02-05

**Authors:** Daniela Melnik, Jayashree Sahana, Thomas J. Corydon, Sascha Kopp, Mohamed Zakaria Nassef, Markus Wehland, Manfred Infanger, Daniela Grimm, Marcus Krüger

**Affiliations:** 1 Clinic for Plastic, Aesthetic and Hand Surgery, Otto von Guericke University, Leipziger Str. 44, 39120 Magdeburg, Germany; daniela.melnik@med.ovgu.de (D.M.); sascha.kopp@med.ovgu.de (S.K.); mohamed.nassef@med.ovgu.de (M.Z.N.); markus.wehland@med.ovgu.de (M.W.); manfred.infanger@med.ovgu.de (M.I.); 2 Department of Biomedicine, Aarhus University, Hoegh-Guldbergsgade 10, 8000 Aarhus C, Denmark; jaysaha@biomed.au.dk (J.S.); corydon@biomed.au.dk (T.J.C.); dgg@biomed.au.dk (D.G.); 3 Department of Ophthalmology, Aarhus University Hospital, Palle Juul-Jensens Boulevard 99, 8200 Aarhus N, Denmark; 4 Research Group “Magdeburger Arbeitsgemeinschaft für Forschung unter Raumfahrt- und Schwerelosigkeitsbedingungen” (MARS), Otto von Guericke University, Universitätsplatz 2, 39106 Magdeburg, Germany; 5 Department of Microgravity and Translational Regenerative Medicine, Otto von Guericke University, Pfälzer Platz, 39106 Magdeburg, Germany

**Keywords:** glucocorticoids, 3D growth, nuclear factor kappa-light-chain-enhancer of activated B-cells (NF-κB), epithelial–mesenchymal transition, anoikis, proliferation

## Abstract

Detachment and the formation of spheroids under microgravity conditions can be observed with various types of intrinsically adherent human cells. In particular, for cancer cells this process mimics metastasis and may provide insights into cancer biology and progression that can be used to identify new drug/target combinations for future therapies. By using the synthetic glucocorticoid dexamethasone (DEX), we were able to suppress spheroid formation in a culture of follicular thyroid cancer (FTC)-133 cells that were exposed to altered gravity conditions on a random positioning machine. DEX inhibited the growth of three-dimensional cell aggregates in a dose-dependent manner. In the first approach, we analyzed the expression of several factors that are known to be involved in key processes of cancer progression such as autocrine signaling, proliferation, epithelial–mesenchymal transition, and anoikis. Wnt/β-catenin signaling and expression patterns of important genes in cancer cell growth and survival, which were further suggested to play a role in three-dimensional aggregation, such as *NFKB2*, *VEGFA*, *CTGF*, *CAV1, BCL2*(*L1*), or *SNAI1*, were clearly affected by DEX. Our data suggest the presence of a more complex regulation network of tumor spheroid formation involving additional signal pathways or individual key players that are also influenced by DEX.

## 1. Introduction

**Figure 1:**
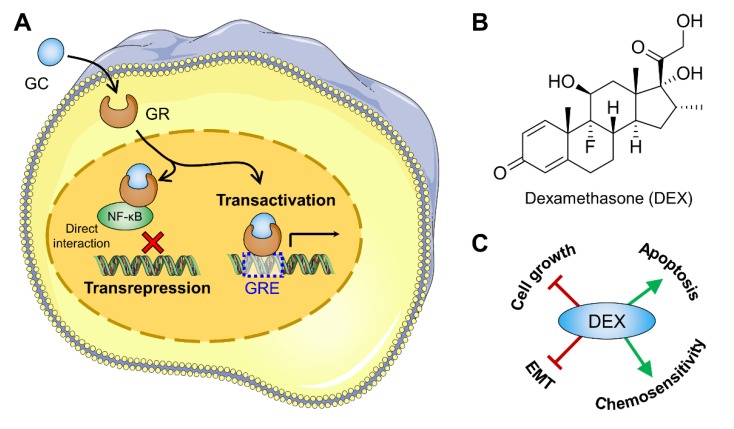
(**A**) Sketch showing the genomic actions of glucocorticoids (GCs) such as dexamethasone (DEX). When bound to DEX, the glucocorticoid receptor (GR) complex translocates into the nucleus and modifies the synthesis of several metabolic proteins. This is done either through directly binding to glucocorticoid response elements (GREs) on the DNA or through influencing the activity of transcription factors (i.e., nuclear factor kappa-light-chain-enhancer of activated B-cells, NF-κB); (**B**) Chemical structure of DEX; (**C**) Described effects of DEX on cancer cells. Parts of the figure are drawn using pictures from Servier Medical Art (https://smart.servier.com), licensed under a Creative Commons Attribution 3.0 Unported License (https://creativecommons.org/licenses/by/3.0).

Glucocorticoids (GCs) are a class of steroid hormones involved in many physiological processes such as metabolism, proliferation, differentiation, and survival of cells [1]. GCs induce their pharmacodynamic effects through binding to glucocorticoid receptors (GRs) [2], which interact downstream with signaling molecules in the cytoplasm or are able to translocate into the nucleus, where they repress the activity of other transcription factors (such as nuclear factor kappa-light-chain-enhancer of activated B-cells, NF-κB, or activator protein 1, AP-1) or initiate transcription of genes associated with anti-inflammatory and immunosuppressive effects (via binding to specific glucocorticoid response elements, GREs) (Figure 1A) [3,4]. Due to these properties, GCs are utilized in the treatment of a variety of immunological disorder treatments to reduce pain and electrolyte imbalance, but also to enhance the anti-tumor effect of chemotherapeutics and prevent adverse effects caused by cytotoxic agents [5,6,7]. The synthetic GC dexamethasone (DEX; Figure 1B) is commonly administered as a supportive care co-medication to reduce cancer-related fatigue in patients with advanced disease [8]. DEX was further reported to inhibit proliferation of different cancer cells in vitro and in vivo [9,10,11,12,13]. Effective inhibition of tumor growth was suggested to be associated with downregulation of JAK3/STAT3, hypoxia inducible factor 1α, vascular endothelial growth factor (VEGF), and interleukin-6 [12,14,15]. Nevertheless, the exact mechanism by which DEX suppresses cancer cell growth is still unclear.

Over the last 50 years, the incidence of thyroid cancer has increased worldwide and the incidence rate is still on the rise. The result of improved diagnostic procedures, an elevated prevalence of individual risk factors (e.g., obesity), and increased exposure to environmental risk factors (e.g., iodine levels), thyroid cancer is the most common form of endocrine malignancy today [16] and is expected to become the fourth leading type of cancer across the globe [17]. Especially poorly differentiated thyroid tumors are aggressive and tend to metastasize. The prognosis for differentiated thyroid cancer is related to the capability of tumor cells to accumulate radioiodine. Due to de-differentiation, some tumor cells may lose their iodine uptake capability, leaving only extremely limited treatment options, despite intensive searches for new drugs and targets. Therefore, novel approaches to control thyroid cancer progression are required.

Metastasis is the most limiting factor in cancer therapy and responsible for 90% of cancer-related deaths [18]. During the development of metastatic competence, carcinoma cells change their adhesive properties, secrete proteinases, and become motile, which allows them to detach from their primary tumor [19]. Therefore, tumor cells respond to mechanical signals, sensed by integrins or other adhesion receptors [20,21], and chemical signals, sensed by chemokines or growth factor receptors [22] causing changes in their transcriptional profile. The process which enables tumor cells to achieve migration and invasion is called epithelial–mesenchymal transition (EMT) and represents a driving force in tumorigenesis [23]. In the course of EMT, essential proteins for epithelial cell–cell adhesion, such as E-cadherin, are downregulated, thus weakening epithelial tissue integrity and polarization of epithelial cell layers [24]. Under normal circumstances, detached epithelial cells undergo apoptosis, a phenomenon termed anoikis. Cancer cells acquire resistance to anoikis to survive after they have left the primary tumor. In this way, they are able to travel via the circulatory and lymphatic systems disseminating throughout the body. EMT and anoikis resistance are critical steps of the metastatic cascade and potential targets to impact a natural molecular prerequisite for the aggressive metastatic spread of cancer [25,26].

Microgravity (µ*g*) has become a powerful tool in cancer research by enabling metastasis-like cell detachment and formation of three-dimensional (3D) multicellular spheroids (MCS) [27,28,29,30]. Experiments in µ*g* contribute to drug discovery by providing an environment which is helpful to detect changes in gene expression and protein synthesis and secretion that occur during the progression from 2D to 3D growth and which might represent new targets for drug development against thyroid cancer. A couple of these proteins were found in follicular thyroid cancer cells by analyzing multiple pilot studies, performed in µ*g*, with the help of semantic methods [31,32]. Some of these potential drugs, including DEX, were recently reviewed [33]. In this study, we investigate the effects of DEX supplementation on the growth of follicular thyroid cancer (FTC) cells exposed to simulated µ*g* produced by a random positioning machine (RPM).

## 2. Materials and Methods

### 2.1. Cell Culture

The human follicular thyroid carcinoma cell line FTC-133 was cultured in RPMI-1640 medium (Life Technologies, Carlsbad, CA, USA), supplemented with 10% fetal calf serum (FCS; Sigma-Aldrich, St. Louis, MO, USA), and 1% penicillin/streptomycin (Life Technologies) at 37 °C and 5% CO_2_ until use for the experiment. For RPM experiments FTC-133 cells were seeded at a density of 1 × 10^6^ cells per flask either in T25 cell culture flasks (Sarstedt, Nümbrecht, Germany) for mRNA and protein extraction or in slide flasks (Sarstedt) for immunofluorescence staining. Cells were given at least 24 h to attach to the bottom of the flasks.

### 2.2. Dexamethasone Treatment

Water-soluble DEX (dexamethasone–cyclodextrin complex) was purchased from Sigma-Aldrich. Then, 24 h after seeding, cells were synchronized in RPMI-1640 medium with 0.25% FCS and 1% penicillin/streptomycin for 4 h. Afterwards, the cells were cultured according to Section 2.1, supplemented with DEX concentrations of 10 nM, 100 nM, or 1000 nM [34].

### 2.3. Random Positioning Machine

The used desktop-RPM (Dutch Space, Leiden, Netherlands) was located in an incubator with 37 °C/5% CO_2_ and operated in real random mode, with a constant angular velocity of 60°/s. Before the run, the flasks were filled up completely and air bubble-free with medium to avoid shear stress. The slide and culture flasks were installed on the prewarmed RPM. After 4 h (short-term experiments) or 3 days (long-term experiments), the cells were photographed and fixed with 4% paraformaldehyde (PFA; Carl Roth, Karlsruhe, Germany) for immunostaining. For RNA and protein extraction adherent cells were harvested by adding ice-cold phosphate-buffered saline (PBS; Life Technologies) and using cell scrapers. The suspensions were centrifuged at 3000× *g* for 10 min at 4 °C followed by discarding the PBS and storage of cell pellets at −150 °C. MCS were collected by centrifuging supernatant at 3000× *g* for 10 min at 4 °C and subsequent storage at −150 °C. Corresponding static controls were prepared in parallel under the same conditions and stored next to the device in an incubator.

### 2.4. Phase Contrast Microscopy

Cells were observed and photographed using an Axiovert 25 Microscope (Carl Zeiss Microscopy, Jena, Germany) equipped with a Canon EOS 550D camera (Canon, Tokio, Japan).

### 2.5. Immunofluorescence Microscopy

Immunofluorescence staining was performed to visualize possible translocal alteration of NF-κB proteins and β-catenin by dexamethasone in cells. The PFA-fixed cells were permeabilized with 0.1% Triton^TM^ X-100 for 15 min and blocked with 3% bovine serum albumin (BSA) for 45 min at ambient temperature. Afterwards, the cells were labeled with primary NF-κB p65 rabbit polyclonal antibody #PA1-186 (Invitrogen, Carlsbad, CA, USA) at 1 µg/mL or β-catenin mouse monoclonal antibody #MA1-300 (Invitrogen) at a dilution of 1:200 in 0.1% BSA and incubated overnight at 4 °C in a moist chamber. The next day, cells were washed three times with PBS before incubation with the secondary Alexa Fluor 488 (AF488)-conjugated anti-rabbit (Cell Signaling Technology, Danvers, MA, USA) or anti-mouse antibody (Invitrogen) at a dilution of 1:1000 for 1 h at ambient temperature. Cells were washed again three times with PBS and mounted with Fluoroshield^TM^ with DAPI (4’,6-diamidino-2-phenylindole) (Sigma-Aldrich). The slides were subsequently investigated with a Zeiss LSM 710 confocal laser scanning microscope (Carl Zeiss) [35].

### 2.6. mRNA Isolation and Quantitative Real-Time PCR

RNA isolation and quantitative real-time PCR were performed according to routine protocols [36,37,38]. Briefly, RNA was isolated by using the RNeasy Mini Kit (Qiagen, Venlo, Netherlands) according to the manufacturer’s protocol and quantified with a spectrophotometer. Afterwards, cDNA was produced with the High Capacity cDNA Reverse Transcription Kit (Applied Biosystems, Foster City, CA, USA) following manufacturer’s instructions. To determine the expression level of the target genes shown in Table S1, quantitative real-time PCR was performed applying the Fast SYBR™ Green Master Mix (Applied Biosystems) and the 7500 Fast Real-Time PCR System (Applied Biosystems). Primers were designed to have a T_m_ ≈ 60 °C and to span exon/exon boundaries using Primer-BLAST [39] (Supplementary Materials). Primer were synthesized by TIB Molbio (Berlin, Germany). All samples were measured in triplicates and analyzed by the comparative threshold cycle (*ΔΔC_T_*) method with 18S rRNA as reference.

### 2.7. Western Blot Analysis

Western blot analysis was performed with routine protocols as described previously [36]. The control and DEX-treated samples were collected after 4 h and 3 days, solubilized in lysis buffer and compared to the control samples without DEX. Each condition included three batches with a total number of 24 samples (4 h) and 33 samples (3 days), respectively. Following lysis and centrifugation, aliquots of 40 µg total protein were subjected to SDS-PAGE and Western blotting. The samples were loaded onto Criterion XT 4–12% precast gels (Bio-Rad, Hercules, CA, USA), run for 1 h at 150 V and transferred to a polyvinylidene difluoride membrane using TurboBlot (Bio-Rad) (100 V, 30 min). Cyclophilin B was used as a loading control. Membranes were blocked with TBS-T containing 0.3% I-Block (Applied Biosystems) for 2 h at ambient temperature. For detection of the proteins shown in Table S2, the membranes were incubated overnight at 4 °C in TBS-T containing 0.3% I-Block solutions of the antibodies. The next day, membranes were washed three times with TBS-T for 5 min and incubated for 2 h at ambient temperature with a horseradish peroxidase (HRP)-linked antibody (Cell Signaling Technology) diluted 1:1000 in TBS-T with 0.3% I-Block. The respective protein bands were visualized using Clarity ECL Western Blot Substrate (Bio-Rad). Images were captured with Image Quant LAS 4000 mini (GE Healthcare, Chicago, IL, USA) and analyzed using ImageJ software (imagej.net) for densitometric quantification.

### 2.8. Terminal Deoxynucleotidyl Transferase dUTP Nick-End Labeling (TUNEL) Assay

The Click-iT™ Plus TUNEL assay (Invitrogen) was used for apoptosis detection. FTC-133 cells were cultured in slide flasks (Sarstedt) under static culture conditions or exposed to the RPM, supplemented without or with 1000 nM DEX. After 4 h or 3 days cells were fixed with 4% PFA and prepared for the evaluation of apoptosis. The staining procedure was performed according to the manufacturer’s recommendation. A positive control sample was treated with DNase I (Epicentre, Madison, WI, USA) to induce DNA fragmentation. The stained cells were examined using a Zeiss LSM 800 confocal laser scanning microscope (Carl Zeiss) equipped with an external light source and an objective with a calibrated 630× magnification.

### 2.9. Ki-67 Proliferation Assay

Cells were cultured and prepared as described in Section 2.5. Cells were labeled with an AF488 recombinant anti-Ki-67 antibody #ab197234 (Abcam, Cambridge, UK) at a dilution of 1:100 in 0.1% BSA and incubated overnight at 4 °C. The next day, cells were washed three times with PBS and mounted with Fluoroshield™ with DAPI (Sigma-Aldrich). The cell proliferation was evaluated by a Zeiss LSM 800 confocal laser scanning microscope (Carl Zeiss) and an objective with a calibrated 230× magnification. Five microscopic images for each condition were analyzed using ImageJ (imagej.net). The percentage of Ki-67 positive cells was counted for each condition and normalized to the control.

### 2.10. Spheroid Formation Assay

Approximately 1 × 10^6^ cells per flasks were seeded into T25 cell culture flasks (Sarstedt). After 24 h the culture flasks were filled up completely (air bubble-free) with media and were installed on the prewarmed RPM (37 °C, 5% CO_2_). To investigate the ability of MCS formation, two RPM running time points were considered: media was completely removed from culture flasks after 24 h and after 48 h exposure to the RPM. Flasks were re-filled with fresh media for a further 24 h run on the RPM. After each run, cells were examined and photographed.

### 2.11. Statistics

Statistical evaluation was performed using GraphPad Prism 7.01 (GraphPad Software, San Diego, CA, USA). The nonparametric Mann–Whitney U test was used to compare DEX-free with DEX-treated samples as well as static and µ*g* conditions. All data are presented as mean ± standard deviation (SD) with a significance level of *p* < 0.05.

## 3. Results

**Figure 2:**
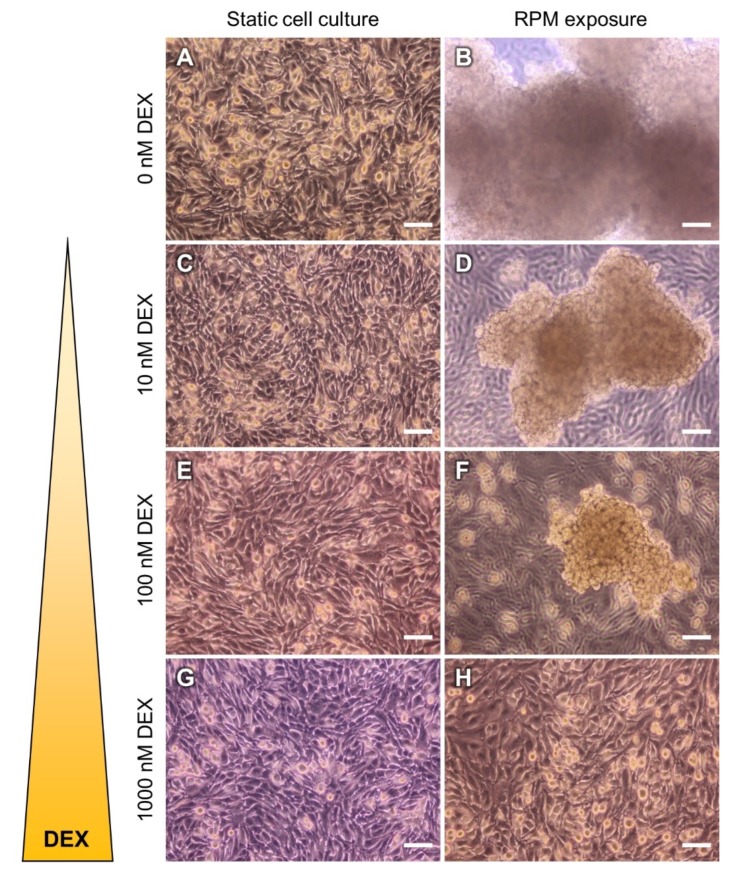
Impact of DEX on the spheroid formation ability of follicular thyroid cancer (FTC)-133 cells exposed to a random positioning machine (RPM). (**A,B**) After three days cells showed a dose-dependent inhibition of spheroid formation when treated with (**C,D**) 10 nM DEX, (**E,F**) 100 nM DEX, or (**G,H**) 1000 nM DEX on the RPM (right column). Scale bars: 100 µm.

Based on the knowledge that NF-κB seems to play a crucial role in spheroid formation of MCF-7 breast cancer cells [34] and that NF-κB subunit p65 (RelA) accumulates in thyroid cancer cells on the RPM [40], we decided to target RelA in µ*g*-grown thyroid cancer cells using DEX. Therefore, we cultured the human follicular thyroid cancer cell line FTC-133 on an RPM in the presence of three different DEX concentrations (10, 100, 1000 nM). After three days on the RPM, the cells showed a DEX dose-dependent inhibition of spheroid formation in µ*g* (Figure 2).

### 3.1. NF-κB Pathway

NF-κB transcription factors play a fundamental role in the tumorigenesis of many cancer types, including thyroid cancer [41,42] and may be a target in the treatment of advanced thyroid cancer [43]. DEX is known to have inhibitory effects on the NF-κB pathway [44].

**Figure 3:**
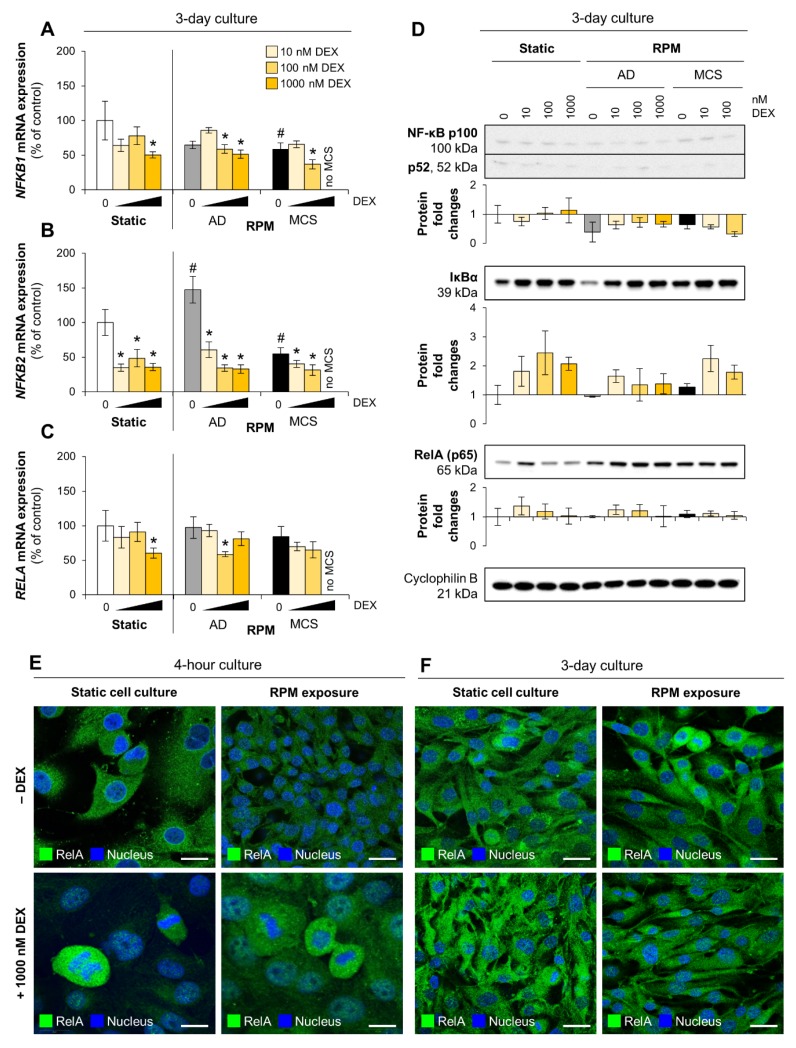
Effect of DEX on NF-κB family members in FTC-133 cells. (**A**) *NFKB1* mRNA expression; (**B**) *NFKB2* mRNA expression; (**C**) *RELA* mRNA expression. Depicted are means of relative mRNA levels ± standard deviations (*n* = 5). *: *p* < 0.05 vs. DEX-free samples. ^#^: *p* < 0.05 vs. static cultures; (**D**) Western blots indicate protein levels of regulated genes after three days. Representatives of each of the three replicates are shown. Diagrams describe relative fold changes to control. AD: adherently growing cells; MCS: multicellular spheroids. (**E**,**F**) Immunofluorescence shows only minor translocation of RelA (green) into the nucleus (blue; 4’,6-diamidino-2-phenylindole (DAPI)-stained) in FTC-133 cells. Scale bars: 20 µm.

We investigated the transcription of the NF-κB family members subunit p50 and its precursor p105 (encoded by *NFKB1*) as well as subunit p52 and its precursor p100 (encoded by *NFKB2*). The mRNA levels of both genes were reduced in MCS cells grown for three days on the RPM and *NFKB2* was upregulated in adherently growing (AD) cells, harvested from the RPM (Figure 3A,B). In addition, we found a dose-dependent inhibitory effect of DEX on the mRNA synthesis of *NFKB2* (Figure 3B) and a less pronounced effect on the mRNA synthesis of *NFKB1* (Figure 3A). In contrast to DEX-treated MCF-7 cells [34], RelA was not translocated into the nucleus of FTC-133 cells in a significant amount after DEX supplementation (Figure 3E,F). Furthermore, RelA expression was not significantly altered by DEX on the transcriptional level (Figure 3C). RelA protein was increased after three days on the RPM and seemed to be augmented by DEX treatment in µ*g*-exposed cells (Figure 3D).

**Figure 4:**
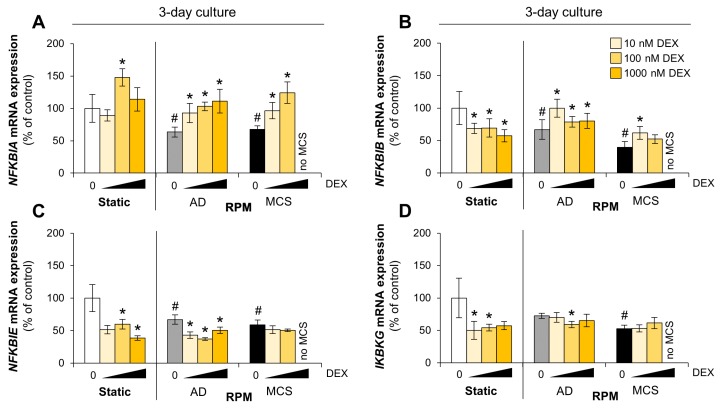
Effect of DEX on NF-κB regulators in FTC-133 cells. (**A**) *NFKBIA* mRNA expression; (**B**) *NFKBIB* mRNA expression; (**C**) *NFKBIE* mRNA expression; (**D**) *IKBKG* mRNA expression. Depicted are means of relative mRNA levels ± standard deviations (*n* = 5). *: *p* < 0.05 vs. DEX-free samples. ^#^: *p* < 0.05 vs. static cultures. AD: adherently growing cells; MCS: multicellular spheroids.

NF-κB dimers can be sequestered in the cytoplasm by the inhibitor of κB (IκB) proteins. Therefore, we analyzed the expression of IκBα (encoded by *NFKBIA*), IκBβ (encoded by *NFKBIB*), and IκBε (encoded by *NFKBIE*). The effect of DEX supplementation on *NFKBIA* expression was limited to RPM-exposed cells (Figure 4A), but it tended to upregulate the IκBα protein level in three-day cultures, independent of gravity (Figure 3D). In addition, DEX lowered *NFKBIB* and *NFKBIE* mRNA in cells cultured under normal conditions. The *NFKBIB* mRNA synthesis seemed to be increased (Figure 4B) whereas *NFKBIE* mRNA synthesis was reduced in adherently growing cells on the RPM (Figure 4C). Overall, the mRNA synthesis of all IκB proteins was reduced by long-term exposure to the RPM.

Activation of NF-κB is initiated by the signal-induced degradation of IκB proteins, mainly via activation of IκB kinase (IKK). IKK is composed of the catalytic IKKα/IKKβ heterodimer and the master regulator NEMO (NF-κB essential modulator), also referred as IKKγ (encoded by *IKBKG*). Three-day MCS showed a reduction in *IKBKG* mRNA synthesis. DEX reduced *IKBKG* mRNA synthesis only in static cultured cells after three days. Under all other conditions, transcription was unaffected by DEX supplementation (Figure 4D).

Since NF-κB is obviously not the main target of DEX in suppressing µ*g*-based spheroid formation of FTC-133 cells, we proceeded to illuminate further cancer-related processes which have been reported in connection with DEX (Figure 1C) and that are also involved in the formation of tumor spheroids.

### 3.2. Growth Factors and Proliferation

**Figure 5:**
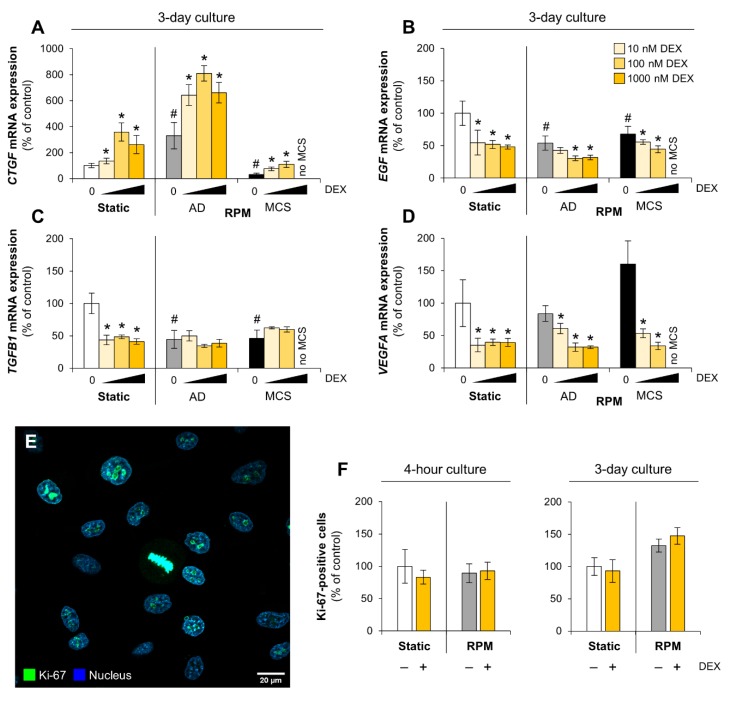
Effect of DEX on autocrine growth factors and proliferation markers in FTC-133 cells. (**A**) *CTGF* mRNA expression; (**B**) *EGF* mRNA expression; (**C**) *TGFB1* mRNA expression; (**D**) *VEGFA* mRNA expression. Depicted are means of relative mRNA levels ± standard deviations (*n* = 5); (**E**) Immunofluorescence. Nuclear expression of Ki-67 indicates proliferating cells; (**F**) Proliferation analysis using Ki-67. *: *p* < 0.05 vs. DEX-free samples. ^#^: *p* < 0.05 vs. static cultures. AD: adherently growing cells; MCS: multicellular spheroids.

Different growth factors are expressed and secreted by cancer cells and contribute to proliferation, survival, and migration. Previous experiments designed to elucidate the growth behavior of cancer cells in µ*g* reveal that especially connective tissue growth factor (CTGF), epidermal growth factor (EGF), transforming growth factor beta (TGF-β), and VEGF were regulated in FTC-133 cells after gravity was omitted [37]. CTGF is a member of the CCN family of secreted, matrix-associated proteins that plays a key role in tumor development, progression, and angiogenesis [45]. CTGF is suggested to regulate cancer cell migration, invasion, angiogenesis, and anoikis [46]. In our experiments, CTGF was upregulated in adherently growing FTC-133 cells after DEX supplementation (Figure 5A). RPM-exposure also enhanced the *CTGF* mRNA level resulting in an additive effect of µ*g* and DEX supplementation. However, the transcription was lower in MCS cells compared to cells in static cultures after three days (Figure 5A).

TGF-β and EGF represent two physiological regulators of thyroid cell differentiation and proliferation. Whereas EGF is a strong mitogen for follicular thyroid cells [47], TGF-β has a complicated role in cancer. Initially, TGF-β is a tumor suppressor that inhibits the growth of thyrocytes and induces apoptosis [48]. However, at later stages of tumor progression, TGF-β acts as a potent EMT inducer and then it plays a fundamental role in tumor progression and metastasis formation [49,50,51]. *EGF* mRNA was downregulated both in presence of DEX and in µ*g* (Figure 5B). *TGFB1* mRNA levels were also lower in µ*g*-grown cells, but DEX decreased *TGFB1* mRNA synthesis only in cells from static cultures (Figure 5C). In addition, DEX suppressed VEGF under normal culture conditions and in both cell populations on the RPM (Figure 5D). In accordance with previous studies that investigated other follicular thyroid cancer cells on the RPM [52], *VEGFA* expression was somewhat increased in MCS cells after three days.

DEX was previously reported to have anti-proliferative effects on human medullary thyroid cancer cells [53]. To prove this effect with follicular thyroid cancer cells and in the context of µ*g*, we searched for cellular markers for proliferation such as the Ki-67 protein (encoded by *MKI67*) [54]. Ki-67 can be detected during all active phases of the cell cycle (G1, S, G2, and M), but not in resting cells (G0). Thus, the nuclear expression of Ki-67 can be evaluated to study tumor proliferation using immunofluorescence microscopy (Figure 5E). In our experiments, neither µ*g* nor DEX had a significant influence on the proliferation of FTC-133 cells (Figure 5F).

### 3.3. Epithelial and Mesenchymal Characteristics, Wnt/β-catenin Signaling

**Figure 6:**
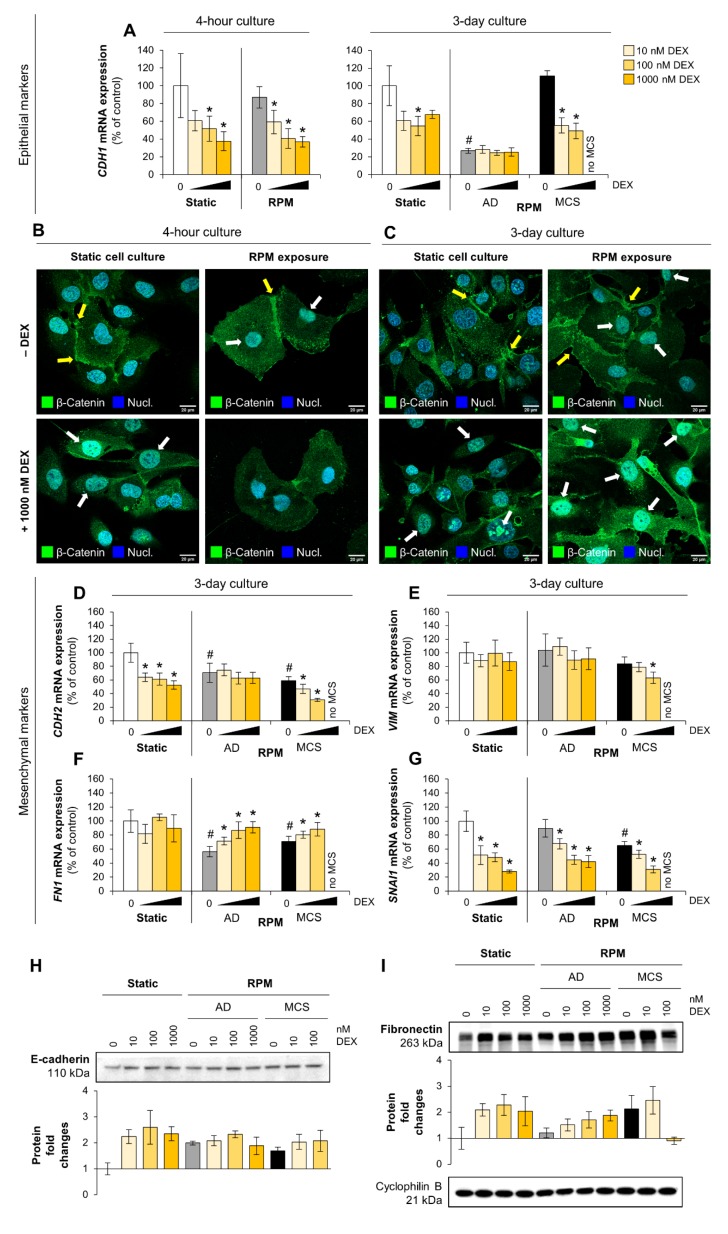
Effect of DEX on the mRNA synthesis of epithelial markers, mesenchymal markers, and other epithelial–mesenchymal transition (EMT) players in FTC-133 cells. (**A**) *CDH1* mRNA expression; (**B,C**) Immunofluorescence. White arrows show translocation of β-catenin (green) into the nucleus (blue; DAPI-stained) both in µ*g* and in the presence of DEX. Yellow arrows indicate an increased occurrence of β-catenin on the plasma membrane in the absence of DEX. Scale bars: 20 µm; (**D**) *CDH2* mRNA expression; (**E**) *VIM* mRNA expression; (**F**) *FN1* mRNA expression; (**G**) *SNAI1* mRNA expression. Depicted are means of relative mRNA levels ± standard deviations (*n* = 5). *: *p* < 0.05 vs. DEX-free samples. ^#^: *p* < 0.05 vs. static cultures; (**H,I**) Western blots indicate protein levels of regulated genes after 3 days. Representatives of each of the three replicates is shown. Diagrams describe relative fold changes to control. AD: adherently growing cells; MCS: multicellular spheroids.

To find signs for EMT, that is also influenced by µ*g* in cancer cells [55], different epithelial (E-cadherin) and mesenchymal markers (N-cadherin, vimentin, fibronectin, Snail1) were analyzed. In a four-hour culture the E-cadherin mRNA (encoded by *CDH1*) was reduced in cells incubated with DEX (Figure 6A), without significant changes in E-cadherin protein levels (Figure 6H). After three days on the RPM, we found a difference in the *CDH1* gene expression between the two phenotypes: adherently growing cells showed a lower, whereas MCS showed a higher, *CDH1* expression compared to control cells. The elevated *CDH1* expression in spheroids was significantly reduced by DEX supplementation (Figure 6A).

The amount of E-cadherin protein was slightly higher in cells exposed to the RPM than those from static cell cultures and was not influenced by DEX in µ*g*. Under normal culture conditions DEX seemed to increase E-cadherin levels (Figure 6H). Downstream of the cadherin complex, β-catenin mRNA (encoded by *CTNNB1*) was not influenced significantly by DEX (Figure S3). However, β-catenin was translocated from the plasma membrane into the nucleus in the presence of DEX (Figure 6B,C) suggesting an involvement of the Wnt/β-catenin pathway. This is supported by the fact that the transcription of the E-cadherin repressor Snail1 (encoded by *SNAI1*) was also downregulated after DEX treatment (Figure 6G).

N-cadherin (encoded by *CDH2*) and vimentin (encoded by *VIM*) were identified to promote thyroid tumorigenesis [56,57]. *CDH2* mRNA was upregulated in cells after short-term exposure (Figure S3C) and downregulated after long-term exposure to the RPM (Figure 6D). Similar to *CDH1, CDH2* expression in spheroids was reduced by DEX supplementation (Figure 6D). Furthermore, DEX elicited the same reducing effects in control cells of a three-day culture. However, adherently growing cells on the RPM were not influenced by DEX. *VIM* expression was not altered by RPM-exposure, but slightly reduced by high DEX concentrations in MCS cells after three days (Figure 6E). RPM-exposure reduced *FN1* mRNA levels in three-day cultures. This effect could be reversed in the presence of DEX (Figure 6F). Protein levels of fibronectin were also slightly increased after DEX supplementation (Figure 6I).

### 3.4. Anoikis Factors

**Figure 7:**
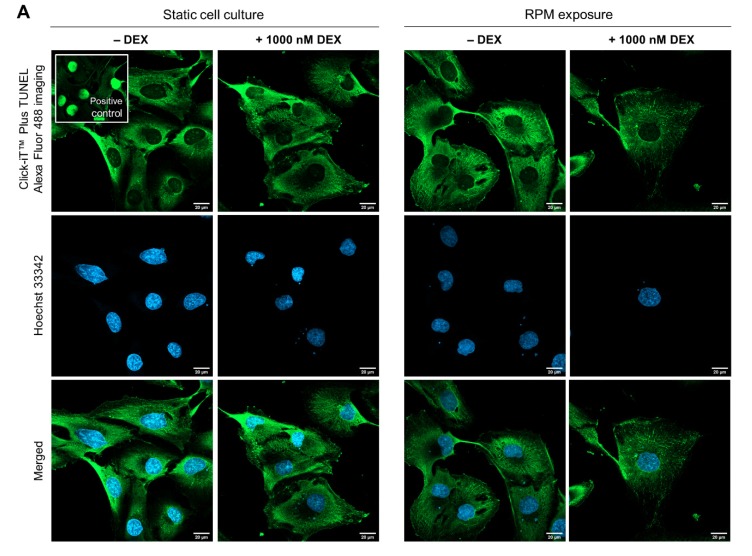

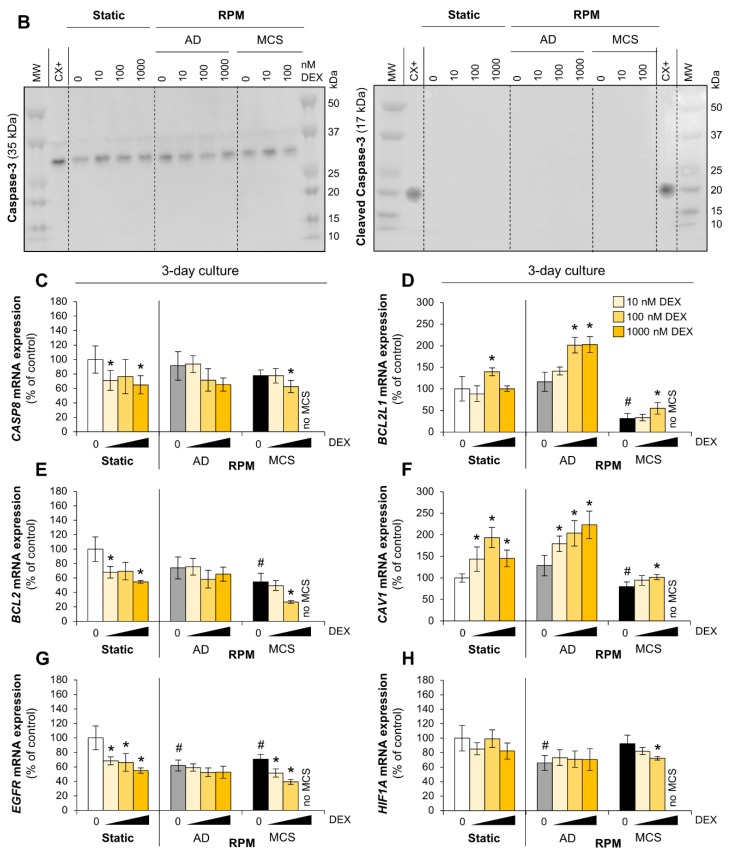
Effect of DEX on apoptosis and anoikis-related proteins in FTC-133 cells. (**A**) No apoptotic cells (green nuclei) were detected by transferase dUTP nick-end labeling (TUNEL) staining after three days. The staining indicates free fluorophores in the cytoplasm in all images except for the positive control. Scale bars: 20 µm; (**B**) Caspase-3 cleavage as an indicator of apoptosis; (**C**) *CASP8* mRNA expression; (**D**) *BCL2L1* mRNA expression; (**E**) *BCL2* mRNA expression; (**F**) *CAV1* mRNA expression; (**G**) *EGFR* mRNA expression; (**H**) *HIF1A* mRNA expression. Depicted are means of relative mRNA levels ± standard deviations (*n* = 5). *: *p* < 0.05 vs. DEX-free samples. ^#^: *p* < 0.05 vs. static cultures. AD: adherently growing cells; MCS: multicellular spheroids.

There is a further possibility that RPM-based spheroid formation of FTC-133 cells in the presence of DEX is abolished through anoikis of detached cells. Cells undergo apoptosis before aggregates are formed. Unfortunately, live/dead staining of detached cells inside the RPM is technically not possible. Adherent cells showed no signs of apoptosis after DEX treatment and after µ*g*-exposure as visualized by a TUNEL staining (Figure 7A). Caspase-3 cleavage tests were negative, both for adherent and spheroid cells in the presence of DEX (Figure 7B). Additionally, we investigated several factors involved in anoikis on the transcriptional level. The cysteine protease caspase-8 (encoded by *CASP8*) is implicated in apoptosis and involved in the induction of NF-κB nuclear translocation [58]. DEX had only a minor effect on *CASP8* gene expression in our experiments (Figure 7C).

The anti-apoptotic protein B-cell lymphoma-extra large (Bcl-xL; encoded by *BCL2L1*) has been implicated in the survival of cancer cells by inhibiting the function of the tumor suppressor p53 [59,60]. *BCL2L1* mRNA synthesis was upregulated in FTC-133 cells exposed to the RPM after four hours and reduced by DEX supplementation (Figure S4C). After three days, the *BCL2L1* mRNA synthesis was downregulated in MCS cells, but remained unchanged in adherently growing cells on the RPM. DEX increased *BCL2L1* mRNA after long-term exposure to the RPM (Figure 7D). In contrast, B-cell lymphoma 2 (Bcl-2; encoded by *BCL2*) was further downregulated by DEX in MCS cells (Figure 7E).

A further factor, caveolin-1 (encoded by *CAV1*), was shown to inhibit anchorage-independent growth, anoikis, and invasiveness in human breast cancer cells [61]. Indeed, µ*g* affected the *CAV1* gene expression during spheroid formation: in MCS cells, the *CAV1* mRNA level was reduced. DEX treatment led to an upregulation of caveolin-1 mRNA (Figure 7F).

The loss of coupling between normal integrin and EGF receptor (EGFR) signaling may be further cause for anoikis resistance in tumor cells [62]. We analyzed *EGFR* mRNA synthesis and found a downregulation of EGFR in RPM-grown cells. In addition, DEX decreased *EGFR* transcription in control cells and MCS after three days (Figure 7G).

Hypoxia inducible factor-1 alpha (HIF-1α; encoded by *HIF1A*) is abundantly expressed in most human carcinomas and their metastases. HIF-1α can be induced via EGFR activation and is known to control central metastasis-associated pathways such as angiogenesis, invasion, and resistance to anoikis [63]. Transcription of *HIF1A* was only downregulated in adherently growing cells in three-day RPM cultures and remained unaffected in MCS or by DEX supplementation (Figure 7H).

### 3.5. Dexamethasone vs. Microgravity—Elucidation of Spheroid Formation Capability

**Figure 8:**
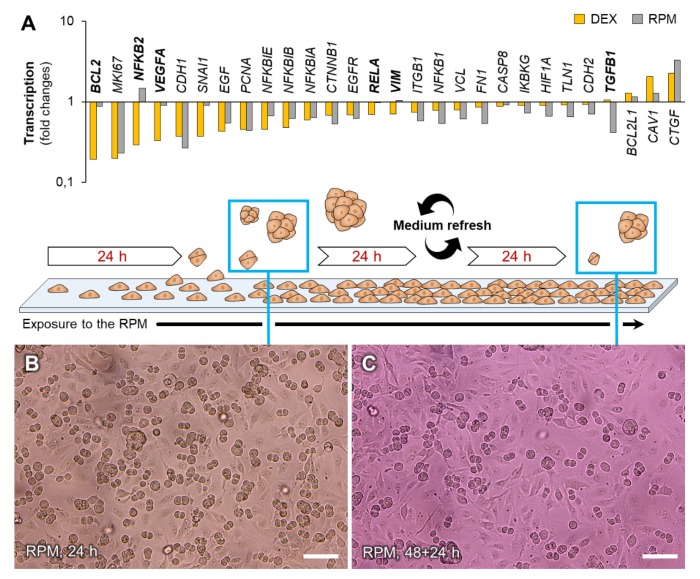
Spheroid formation capability of FTC-133 cells cultured on the RPM. (**A**) Comparison of transcription regulation patterns 4 h after DEX supplementation (yellow bars) and after a three-day RPM-exposure (grey bars). Bold gene symbols indicate fold changes >2.5 or regulation in opposite directions. (**B**) Cells 24 h after the RPM-experiment started; (**C**) Cells 24 h after an initial two-day RPM exposure. Medium was refreshed and spheroids were discarded after the first two days. Although many genes were similarly regulated after DEX supplementation and after a three-day-exposure to the RPM, in contrast to DEX treatment, cells did not lose the ability to form spheroids in µ*g*. Scale bars: 100 µm.

Comparing DEX-induced gene expression data of control cells and transcriptional adaption of FTC-133 cells to µ*g* revealed similar regulation patterns (Figure 8A). We performed an additional two-step RPM culture experiment to check if spheroid formation capability was lost after long-term exposure to µ*g*. Cells that were pre-incubated on the RPM for 48 h showed only marginally reduced spheroid formation during the following 24 h (Figure 8B,C).

## 4. Discussion

We investigated the effects of DEX supplementation on the growth of follicular thyroid cancer cells exposed to simulated µ*g*. During a three-day culture on an RPM, cells grew into the form of a large MCS, as it was reported and studied earlier [28,52,64,65]. Previous research revealed that the addition of DEX to spinner flask cultures led to smaller, irregularly shaped spheroids of rat hepatocytes. Higher DEX concentrations inhibited MCS aggregation and promoted MCS disassembly in culture dishes [66]. Kopp et al. [34] described an inhibitory effect of DEX on the MCS formation rate of MCF-7 breast cancer cells cultured on the RPM. However, the authors did not perform any further analyses to elucidate the underlying effects of DEX on MCF-7 cells. After the current study we can confirm similar effects on FTC-133 cells. We found a dose-dependent inhibition of RPM-based spheroid formation by DEX, that was independent from RelA nuclear translocation which was described for DEX-treated breast cancer cells [34]. This finding agrees with the theory of Bauerle et al. [43] that global regulation of thyroid cancer cell growth is not achieved by NF-κB signaling alone and indicates that NF-κB (pathway) may not be the main target of DEX inhibiting the 3D growth of FTC-133 cells in µ*g*. Therefore, we used transcriptional and translational methods to find answers for the changed growth behavior. Interestingly, after DEX supplementation a couple of genes were regulated in the same direction as after a three-day exposure to the RPM. Since the ability of spheroid formation was not suppressed in the RPM-cultures, especially those genes that are of interest, which had a differential expression pattern (Table 1).

### 4.1. Cell Detachment in Microgravity and Epithelial–Mesenchymal Transition

The EMT describes a fundamental process of cancer progression when carcinoma cells lose their epithelial characteristics and acquire a migratory behavior, indicated by mesenchymal markers. This alteration enables them to escape from their epithelial cell community and invade into surrounding tissues, even at distant locations, and contributes to the acquisition and maintenance of stem cell-like properties [49,67]. In previous studies, DEX proved to suppress cell invasion in bladder cancer [68], inhibited hypoxia-induced EMT in colon cancer cells [69], and reduced TGF-β-induced EMT in non-malignant cells [70].

The interaction of DEX-bound GRs with NF-κB affects the expression of several target genes, one of which is TGF-β [71]. TGF-β induces the upregulation mesenchymal markers such as vimentin and downregulation of the epithelial marker E-cadherin, which are considered critical prerequisites for metastasis in numerous human cancers [72]. Therefore, it is not surprising that the expression of E-cadherin and β-catenin in thyroid cancer is associated with better prognosis [73]. Among the set of analyzed genes, *TGFB1* was regulated differently in µ*g* and in the presence of DEX. Hinz [74] suggested that the TGF-β complex functions as an extracellular mechanosensory that can be activated by contractile forces that are transmitted by integrins. Indeed, µ*g* was identified as a possible cause changing TGF-β expression levels [75]. In four-hour cultures stacked on the RPM, the *TGFB1* mRNA was slightly elevated whereas in three-day cultures the mRNA level was attenuated, maybe due to missing forces in µ*g*. DEX supplementation led to a slow downregulation of *TGFB1* expression in static cell cultures or in follicular thyroid cancer cells grown for four hours on the RPM (Figure S2C). This observation could be cell-type specific, as DEX increased *TGFB1* expression in prostate cancer and pancreatic ductal carcinoma cells [76,77]. TGF-β signaling is identified as one of the most altered pathways in ovarian tumor spheroids [78] and cell aggregation proved to be induced by TGF-β in ovarian cancer cells [79]. Therefore, *TGFB1* could be a possible target gene for DEX that may inhibit spheroid formation of FTC-133 cells in an early culture stage at least in part by downregulation of *TGFB1*.

The translocation of β-catenin into the nucleus as well as upregulation of *FN1* mRNAs suggest an activation of the Wnt/β-catenin pathway after DEX supplementation. On the other hand, expression of Snail1 is reduced in the presence of DEX. However, in ovarian adenocarcinoma cells Snail1 is downregulated when TGF-β and Wnt signaling pathways are co-activated [80]. Snail1 acts as an EMT inducer and a potent repressor of E-cadherin [81,82]. The finding that E-cadherin expression correlates with spheroid formation capability suggests that intercellular adhesion plays a key role in 3D growth [83,84]. Sahana et al. [85] found that the blocking of E-cadherin activity with antibodies promoted µ*g*-driven spheroid formation of MCF-7 cells. Budding of ovarian cancer spheroids from monolayers correlated with the expression of vimentin and lack of cortical E-cadherin [86]. In our experiments the *CDH1* gene was downregulated in adherent cells but remained nearly unchanged in MCS cells of a three-day RPM culture. E-cadherin protein increased after DEX supplementation. This finding is consistent with the observations of Sahana et al. [85] and suggests a quantity-dependent influence of E-cadherin on cancer cell aggregation, that is not directly related to its mRNA synthesis. For renal cell carcinoma, N-cadherin was shown not to be an essential molecule for spheroid formation indicating a somewhat different role from cell–cell adhesion. However, anti-N-cadherin antibodies inhibit spheroid formation in a renal cell carcinoma cell line that expressed N-cadherin alone [87]. Tsai et al. [88] suggested that N-cadherin might play a role in the formation and maintenance of spheroid core structures. Due to a higher affinity of N-cadherin to form homodimers [89], cells with higher N-cadherin expression aggregate first. Indeed, in the spheroid-inducing environment of the RPM, the *CDH2* gene was upregulated after four hours (Figure S3B). DEX reduced *CDH2* expression in these cells as well as in spheroids after three days. This explains the reduced spheroid formation in the presence of DEX, but on the other hand, it suggests a destabilization of formed spheroids.

Fibronectin was downregulated in µ*g*, but upregulated by DEX, in three-day cultures. Thus, it was the only mesenchymal factor showing a significantly altered regulation after DEX supplementation. Abu-Absi et al. [66] previously reported an increase in fibronectin and collagen III mRNA when rat hepatocytes were cultured in spinner flasks in the presence of DEX and suggested that a modification of the extracellular matrix (ECM) contributes to the changes in morphology. It is further known that DEX treatment significantly increases the strength of cell–ECM adhesion in glioblastoma cells and thus decreases their motility [90]. Robinson et al. [91] confirmed in different experiments that fibronectin matrix assembly plays a key role in cell aggregation and spheroid formation. So, it is very likely that DEX alters the ECM composition of FTC-133 cells, including fibronectin, in a way that they are no longer susceptible to 3D aggregation in µ*g*.

Our data indicate that FTC-133 cells were not shifted to a typical mesenchymal phenotype during spheroid formation on the RPM and the phenotype was not strongly influenced by DEX. However, the results confirm the involvement of the Wnt/β-catenin axis and TGF-β-induced signaling in µ*g*-triggered spheroid formation ability. These pathways were affected by DEX and can regulate some individual adhesion and matrix proteins (e.g., E-cadherin and fibronectin) which are important for detachment and 3D aggregation.

### 4.2. Survival of Detached Cells

At least for adherently growing FTC-133 cells, a cell-based assay showed no apoptotic cells after DEX treatment. For osteoblasts it is known that DEX can cause anoikis, probably due to the decreased integrin β1 expression [92]. In our experiments, indeed we found a downregulation of *ITGB1* transcription in DEX-treated cells as well as lower integrin β1 levels in MCS cells that grew in the presence of DEX (Figure S5).

The induction of anoikis occurs through an interplay of the two apoptotic pathways involving activation of caspase-8 and inhibition of Bcl-2 [26]. Caspase-8 expression was only marginally affected by µ*g* and DEX treatment. We observed a counter-regulation for enhanced *CASP8* mRNA synthesis by DEX in cells after four hours on the RPM (Figure S4A). DEX significantly reduced expression of the anti-apoptotic *BCL2* gene in FTC-133 cells but stimulated the expression of the anti-apoptotic Bcl-xL. An upregulation of Bcl-xL expression by DEX was reported earlier for follicular thyroid cancer cells where it promotes survival [93]. That Bcl-2 plays an important role in the efficacy of DEX was confirmed in a study with myeloma cells where Bcl-2 overexpression was associated with resistance to DEX [94]. Overexpression of Bcl-2 correlates with the progression and metastasis of prostate cancer [95] and was shown to inhibit anoikis at least in intestinal epithelial cells [96]. Looking at apoptosis signaling, there are a some, but not all, indications that anoikis can be induced in FTC-133 cells after DEX treatment.

Apart from the apoptotic pathways, there are other proteins playing important roles in the complex network of survival signaling. It has been reported that inhibition of E-cadherin binding prevented cell–cell aggregation and could induce anoikis in epithelial cells [97,98]. In addition, the overexpression of β-catenin, a downstream regulator of cadherin signaling, resulted in anoikis resistance [99]. Indeed, on the transcriptional level we saw a DEX-mediated downregulation of *CDH1* together with an upregulation of *CTNNB1* in MCS cells.

The integral membrane protein caveolin-1 was identified as an important factor in spheroid formation of thyroid cancer cells in an µ*g* environment [65,100] which further inhibits anchorage-independent growth and anoikis, obviously two independent processes, in MCF-7 breast cancer cells [61]. The *CAV1* gene was downregulated when FTC-133 cells formed MCS on an RPM [100]. We were able to confirm this effect in our experiments. The upregulation of *CAV1* expression in the presence of DEX may suppress caveolin’s yet undefined effects on 3D aggregation of thyroid carcinoma cells. Interestingly, caveolin-1 is overexpressed in other metastatic carcinoma cells where it promotes growth [101,102] and resistance to anoikis [61]. Caveolin-1 controls the stability of focal adhesions and contributes to mechanosensing and adaptation in response to mechanical stimuli including cell detachment [103]. Moreno-Vicente et al. [104] demonstrated that caveolin-1 regulates yes-associated protein activity which in turn modulates pathophysiological processes such as ECM remodeling. The authors suggested that this regulation could determine the onset and progression of tumor development. A possible explanation for suppressed spheroid formation would be that a more rigid ECM inhibits the growth into 3D aggregates.

In summary, we found regulatory indications but no clear evidence of anoikis after DEX treatment, as both caspase-3 cleavage and TUNEL staining were negative. Therefore, we suggest that apoptosis does not play a (major) role in inhibition of FTC-133 spheroid formation by DEX.

### 4.3. Autocrine Signaling

Growth factors have been considered to be involved in spheroid formation of thyroid cancer cells for long time. During the Shenzhou-8 space experiment, extraordinarily large 3D aggregates were formed by FTC-133 cells which showed an altered expression of *EGF* and *CTGF* genes under real µ*g* [105]. A decreased expression of *CTGF* in MCS compared to an increased expression in adherent cells was observed after cultivation of FTC-133 on different µ*g*-devices suggesting an important role for CTGF in spheroid formation [65]. DEX supplementation resulted in an upregulation of *CTGF* mRNA synthesis. In vitro, CTGF was identified to stimulate ECM synthesis, proliferation, or integrin expression and has been implicated in different cancer-related processes, comprising migration, invasion, angiogenesis, and anoikis [46]. Elevated *CTGF* expression levels in primary papillary thyroid carcinoma samples were correlated with metastasis [106]. EGF acts as a strong mitogen for follicular thyroid cells [47] and has been shown to increase the spheroid size in various tumor cell lines [107,108,109]. Both, DEX and µ*g* reduced *EGF* expression. However, CTGF upregulation and EGF downregulation by DEX cannot explain the inhibition of spheroid formation, especially since adherent cells on the RPM are able to form MCS but show the same gene regulations.

VEGF promotes tumor angiogenesis by stimulating proliferation and survival of endothelial cells and can directly modulate cancer cell behavior [110]. Studies have shown that VEGF expression is upregulated in most human tumors and correlates with the risk for the development of metastasis in papillary thyroid cancer [111,112]. VEGF expression can be upregulated in response to hypoxia and was found in the microenvironment of tumor spheroids formed by HT-29 human colon cancer cells [113]. Furthermore, spheroids formed by FTC-133 cells on an RPM showed an increase in *VEGFA* gene expression [52]. Inhibition of VEGF signaling significantly reduced cell viability of thyroid cancer cells and increased apoptosis in the NPA′87 tumor-derived cell line [114]. DEX was reported to reduce VEGF secretion in some head and neck cancer cells via STAT3 [115]. We can confirm a decreasing effect of DEX on *VEGFA* expression in FTC-133 cells, independently of their exposure to µ*g* or their growth behavior on the RPM. A decrease in VEGF-A by treatment using siRNA or anti-VEGF-A, reduced spheroid formation, proliferation, migration, and invasion of epidermal cancer stem cells [116]. This finding highlights the role of VEGF (signaling) in the formation of solid tumors and could also provide an explanation for the effect of DEX on FTC-133 cells. In T47D breast cancer cells, DEX was shown to affect the PI3K/AKT/mTOR pathway [117] that could also be a possible target in thyroid cancer cells responsible for the suppression of spheroid formation [118].

## 5. Conclusions

In our study, DEX suppressed spheroid formation of FTC-133 cells cultured on an RPM in a dose-dependent manner. Interestingly, DEX did not influence NF-κB in a way that would explain the inhibition of µ*g*-triggered spheroid formation indicating that NF-κB (pathway) may not be the main target of DEX in FTC-133 cells. However, transcriptional regulation of important individual factors in cancer cell biology, which were previously suggested to play a role in spheroid formation, was clearly affected by DEX. Thereby, our data indicate the presence of a more complex regulation network of spheroid formation also involving other signal pathways, such as Wnt/β-catenin and TGF-β, that regulate adhesion and matrix proteins which are important for cell detachment and 3D growth. According to our results, it will be necessary to carry out a broad transcriptome analysis in order to identify the exact influence of DEX on the growth behavior of follicular thyroid cancer cells. Furthermore, it needs to be clarified whether DEX not only inhibits formation of spheroids but also promotes their disassembly in µ*g*.

## Figures and Tables

**Table 1:** Significant differences (*p* < 0.05) in mRNA synthesis of adherently growing FTC-133 cells in presence of DEX in static cell culture compared with cells grown without DEX on the RPM.

Process/Pathway	4-Hour Culture	Both Time Points	3-Day Culture
NF-κB pathway	*NFKB1*↑, *NFKBIA*↓, *NFKBIB*↓	*NFKB2*↓, *NFKBIE*↓	*NFKB1*↓, *RELA*↓, *NFKBIA*↑, *IKBKG*↓
Autocrine signaling	*EGF*↓, *TGFB1*↓	*VEGFA*↓	
EMT	*CDH1*↓, *CTNNB1*↓, *VIM*↓,	*CDH2*↓, *SNAI1*↓	*CDH1*↑
Anoikis	*CASP8*↓, *BCL2*↓, *BCL2L1*↓, *EFGR*↓, *HIF1A*↓		*HIF1A*↑
Proliferation	*MKI67*↓, *PCNA*↓		*MKI67*↑

↑: significant upregulation in DEX-treated cells; ↓: significant downregulation in DEX-treated cells.

## Supplementary Materials

The following materials are available online at https://www.mdpi.com/2073-4409/9/2/367/s1, Figure S1. Effect of 4 h DEX treatment on NF-κB family members and regulators in FTC-133 cells; Figure S2. Effect of 4 h DEX treatment on growth factors and proliferation markers in FTC-133 cells. Figure S3: Effect of 4 h DEX treatment on epithelial markers, mesenchymal markers and other EMT players in FTC-133 cells; Figure S4: Effect of 4 h DEX treatment on anoikis-related factors in FTC-133 cells; Figure S5: Effect of DEX on cell–cell contacts of FTC-133 cells.; Table S1: Primer sequences used in the quantitative real-time PCR; Table S2: Antibodies used for Western blot analyses.
