# Supplementary material for: Dexamethasone Inhibits Spheroid Formation of Thyroid Cancer Cells Exposed to Simulated Microgravity
Source: Cells. 2020 Feb 5;9(2):367. doi: 10.3390/cells9020367 (PMC7072698; doi:10.3390/cells9020367)
Supplement: Supplementary file 1 [file cells-09-00367-s001.pdf]

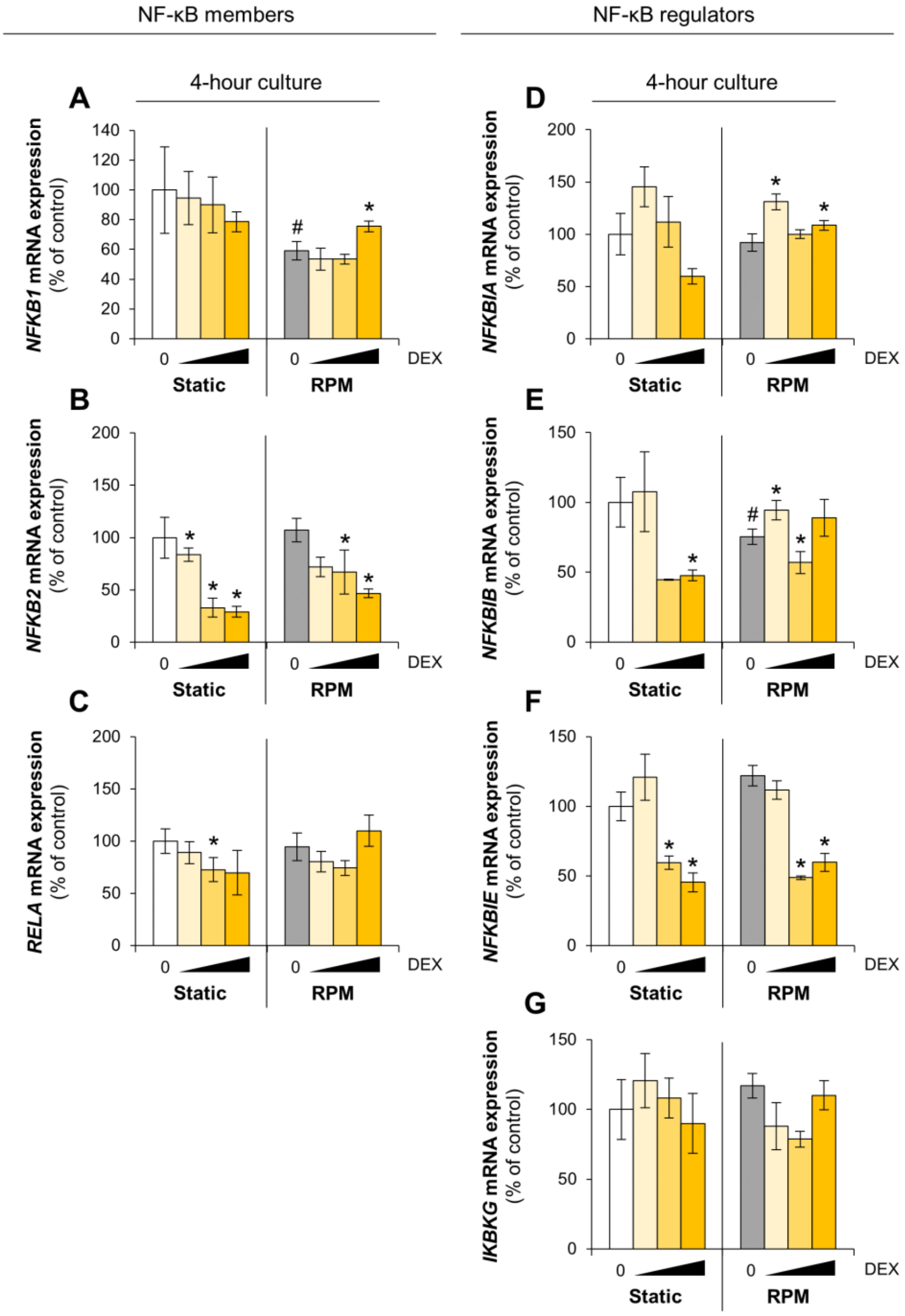

Figure S1. Effect of 4 h DEX treatment on NF-κB family members and regulators in FTC-133 cells. (**A**) *NFKB1* mRNA expression; (**B**) *NFKB2* mRNA expression; (**C**) *RELA* mRNA expression. (**D**) *NFKBIA* mRNA expression; (**E**) *NFKBIB* mRNA expression; (**F**) *NFKBIE* mRNA expression; (**G**) *IKBKG* mRNA expression. Depicted are means of relative mRNA levels ± standard deviations ($n = 5$). *: $p < 0.05$ vs. DEX-free samples. #: $p < 0.05$ vs. static cultures. AD: adherently growing cells; MCS: multicellular spheroids.

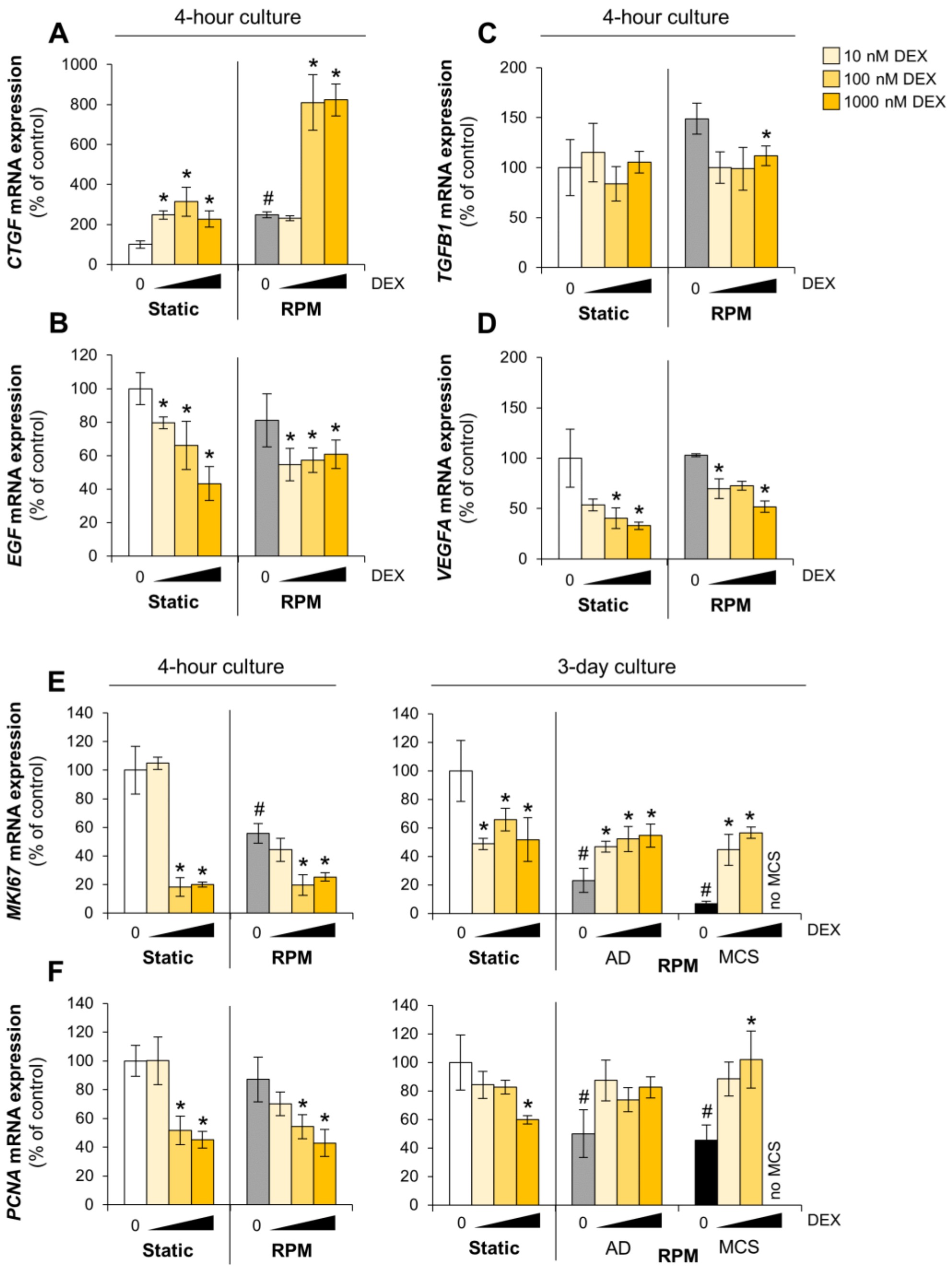

Figure S2. Effect of 4 h DEX treatment on growth factors and proliferation markers in FTC-133 cells. (**A**) *CTGF* mRNA expression; (**B**) *EGF* mRNA expression; (**C**) *TGFB1* mRNA expression; (**D**) *VEGFA* mRNA expression. (**E**) *MKI67* mRNA expression; (**F**) *PCNA* mRNA expression. Depicted are means of relative mRNA levels ± standard deviations ($n = 5$). *: $p < 0.05$ vs. DEX-free samples. #: $p < 0.05$ vs. static cultures. AD: adherently growing cells; MCS: multicellular spheroids.

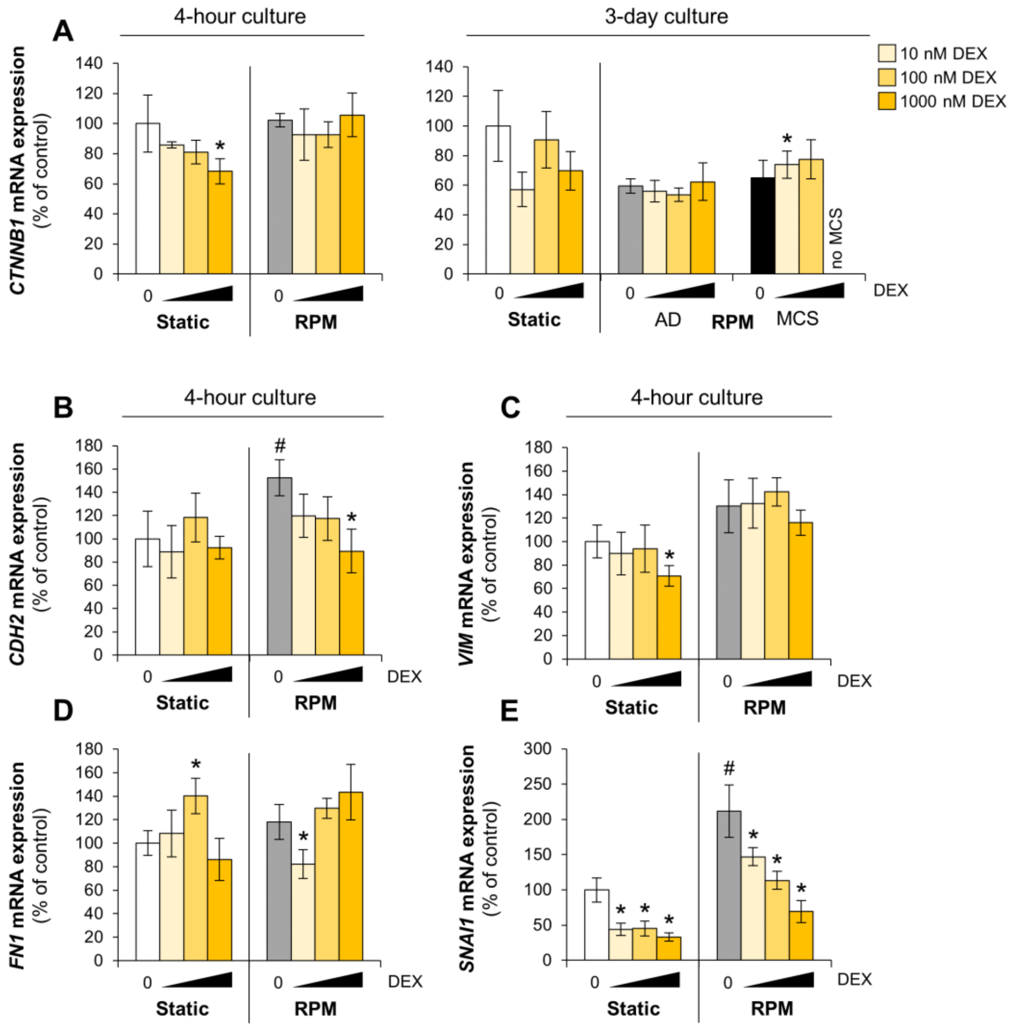

Figure S3. Effect of 4 h DEX treatment on growth factors and proliferation markers in FTC-133 cells. (**A**) *CTGF* mRNA expression; (**B**) *EGF* mRNA expression; (**C**) *TGFB1* mRNA expression; (**D**) *VEGFA* mRNA expression. (**E**) *MKI67* mRNA expression; (**F**) *PCNA* mRNA expression. Depicted are means of relative mRNA levels ± standard deviations ($n = 5$). *: $p < 0.05$ vs. DEX-free samples. #: $p < 0.05$ vs. static cultures. AD: adherently growing cells; MCS: multicellular spheroids.

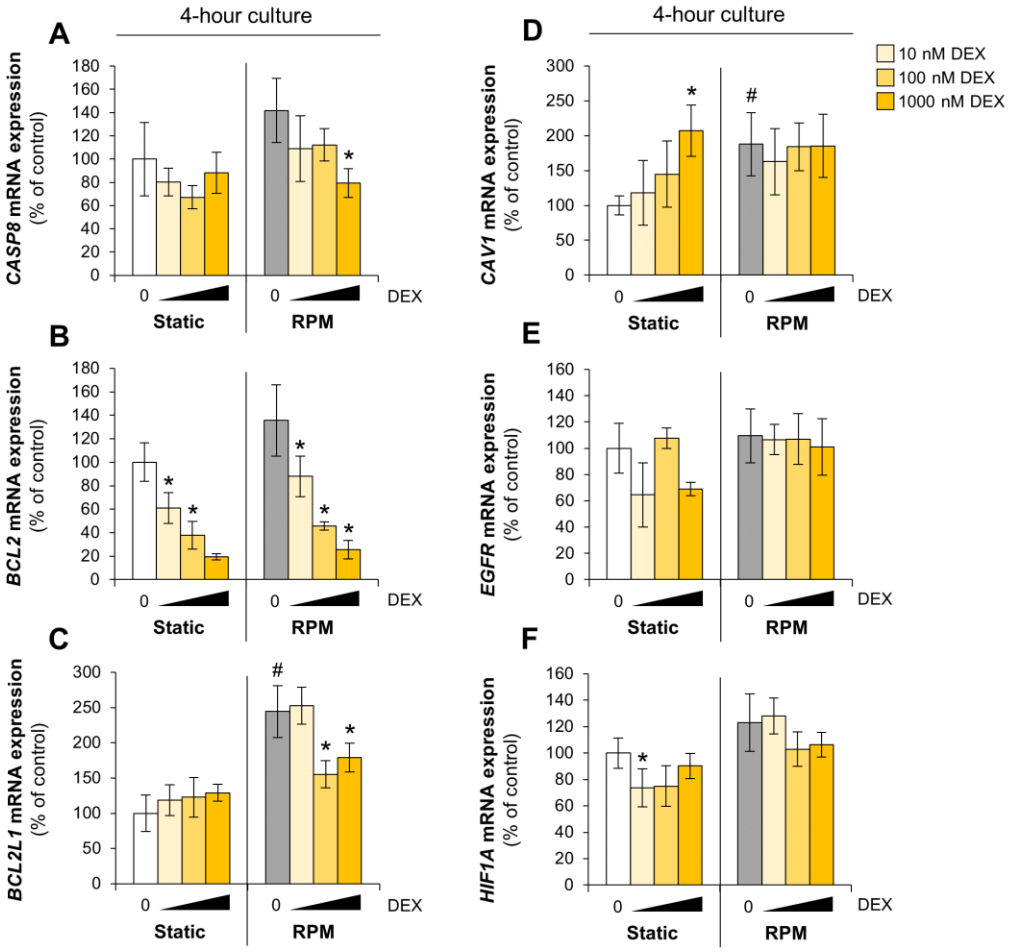

Figure S4. Effect of 4 h DEX treatment on anoikis-related factors in FTC-133 cells. (**A**) *CASP8* mRNA expression; (**B**) *BCL2* mRNA expression; (**C**) *BCL2L1* mRNA expression; (**D**) *CAV1* mRNA expression. (**E**) *EGFR* mRNA expression; (**E**) *HIF1A* mRNA expression. Depicted are means of relative mRNA levels ± standard deviations ($n = 5$). *: $p < 0.05$ vs. DEX-free samples. #: $p < 0.05$ vs. static cultures. AD: adherently growing cells; MCS: multicellular spheroids.

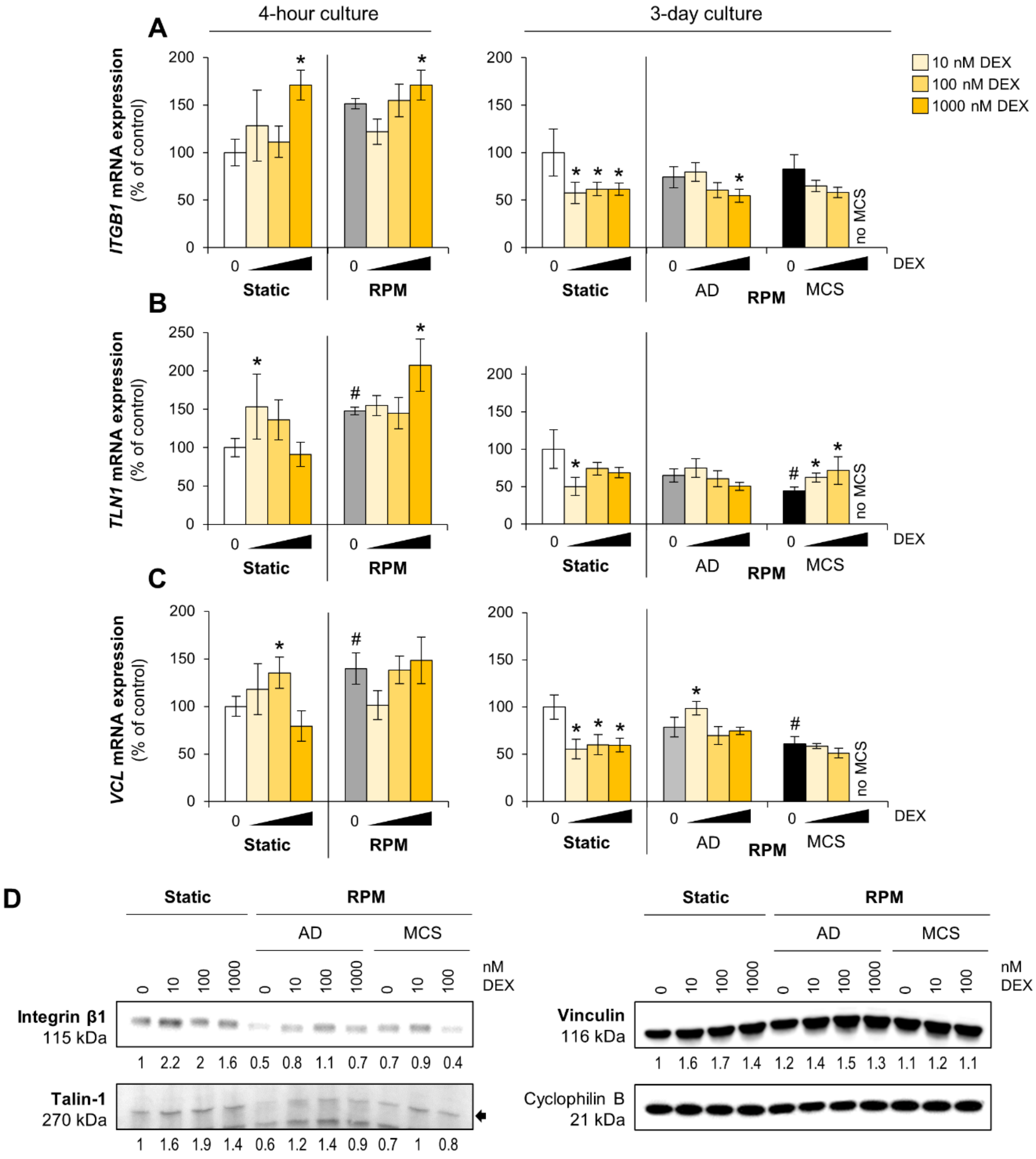

Figure S5. Effect of DEX on cell-cell contacts of FTC-133 cells. (**A**) *ITGB1* mRNA expression; (**B**) *TLN1* mRNA expression; (**C**) *VCL* mRNA expression. Depicted are means of relative mRNA levels ± standard deviations ($n = 5$). *: $p < 0.05$ vs. DEX-free samples. #: $p < 0.05$ vs. static cultures. (**D**) Western blots indicate protein levels of regulated genes after 3 days. Representatives each of three replicates are shown. Numbers describe relative fold changes to control. AD: adherently growing cells; MCS: multicellular spheroids.

Table S1. Primer sequences used in the quantitative real-time PCR.

Gene	Primer Name	Sequence (5′->3′)
18S rRNA	18S-F	GGAGCCTGCGGCTTAATTT
	18S-R	CAACTAAGAACGGCCATGCA
BCL2	BCL2-F	CCTGTGGATGACTGAGTACCTGAA
	BCL2-R	TCAGAGACAGCCAGGAGAAATCA
BCL2L1	BCL2L1-F	CATGGCAGCAGTAAAGCAAG
	BCL2L1-R	TAGAGTTCCACAAAAGTATC
CASP8	CASP8-F	TGCAAAAGCACGGGAGAAAG
	CASP8-R	CTCTTCAAAGGTCGTGGTCAAAG
CAV1	CAV1-EX1-F	CCTCCTCACAGTTTTCATCCA
	CAV1-EX1-R	TGTAGATGTTGCCCTGTTCC
CDH1	CDH1-F	GCTGGACCGAGAGAGTTTCC
	CDH1-R	CAGCTGTTGCTGTTGTGCTT
CDH2	CDH2-F	GTGCATGAAGGACAGCCTCT
	CDH2-R	CCACCTTAAAATCTGCAGGC
CTGF	CTGF-F	ACAAGGGCCTCTTCTGTGACTT
	CTGF-R	GGTACACCGTACCACCGAAGAT
CTNNB1	CTNNB1-F	GAAACAGCTCGTTGTACCGC
	CTNNB1-R	ATCCACTGGTGAACCAAGCA
EGF	EGF-F	TGCCAGCTGCACAAATACAGA
	EGF-R	TCTTACGGAATAGTGGTGGTCATC
EGFR	EGFR-F	TTGCCGCAAAGTGTGTAACG
	EGFR-R	GAGATCGCCACTGATGGAGG
FN1	FN1-F	AGATCTACCTGTACACCTTGAATGACA
	FN1-R	CATGATACCAGCAAGGAATTGG
HIF1A	HIF1A-F	TGCTTTAACTTTGCTGGCCC
	HIF1A-R	AGTTTCTGTGTCGTTGCTGC
IKBKG	IKBKG-F	AACTGGGACTTTCTCGGAGC
	IKBKG-R	GGCAAGGGCTGTCAGCAG
ITGB1	ITGB1-F	GAAAACAGCGCATATCTGGAAATT
	ITGB1-R	CAGCCAATCAGTGATCCACAA
MKI67	KI67-F	TGGGGAAAGTAGGTGTGAAAGAAG
	KI67-R	CTCCTTAAACGTTCTGATGCTCTTG
NFKB1	NFKB1-F	CTTAGGAGGGAGAGCCCAC
	NFKB1-R	TGAAACATTTGTTCAGGCCTTC
NFKB2	NFKB2-F	GTACAAAGATACGCGGACCC
	NFKB2-R	CCAGACCTGGGTTGTAGCA
NFKBIA	NFκBIA-F	AATGCTCAGGAGCCCTGTAAT
	NFκBIA-R	CTGTTGACATCAGCCCCACA
NFKBIB	NFKBIB-F	CCCGGAGGACCTGGGTT
	NFKBIB-R	GCAGTGCCGTGTCCCC
NFKBIE	NFκBIE-F	TGGGCATCTCATCCACTCTG
	NFκBIE-R	ACAAGGGATTCCTCAGTCAGGT
PCNA	PCNA-F	ACTAAAATGCGCCGGCAAT
	PCNA-R	AACTTTCTCCTGGTTTGGTGCTT
RELA	NFκB-F	CGCTTCTTCACACACTGGATTC
	NFκB-R	ACTGCCGGGATGGCTTCT
SNAI1	Snail1-F	CCAGTGCCTCGACCACTATG
	Snail1-R	CTGCTGGAAGGTAAACTCTGGA

TGFB1	TGFB1-F	CACCCGCGTGCTAATGGT
	TGFB1-R	AGAGCAACACGGGTTCAGGTA
TLN1	TLN1-F	GATGGCTATTACTCAGTACAGACAACTGA
	TLN1-R	CATAGTAGACTCCTCATCTCCTTCCA
VCL	VCL-F	GTCTCGGCTGCTCGTATCTT
	VCL-R	GTCCACCAGCCCTGTCATTT
VEGFA	VEGFA-F	GCGCTGATAGACATCCATGAAC
	VEGFA-R	CTACCTCCACCATGCCAAGTG
VIM	VIM-F	TTCAGAGAGAGGAAGCCGAAAAC
	VIM-R	AGATTCCACTTTGCGTTCAAGGT

Table S2. Antibodies used for Western blot analyses.

Antibodies	Company, Product Nr.	Species[1]	MW (kDa)	Dilution
Caspase 3	CST, #9662S	Rb	35	1:800
- cleaved	CST, #9664S	Rb	17	1:500
β-catenin	Invitrogen, #MA1-300	Ms	94	1:500
Cyclophilin B	Abcam, #ab178397	Rb	18	1:1000
E-cadherin	Abcam, #ab1416	Ms	110	1:1000
Fibronectin	Invitrogen, #MA5-11981	Rb	263	1:2000
IκBα	CST, #9242S	Rb	39	1:1000
Integrin β1	CST, #4706S	Ms	115	1:1000
NF-κB p52	Invitrogen, #MA5-15110	Rb	52, 100	1:1000
NF-κB p65	Sigma, #SAB4502615	Rb	65	1:1000
Talin	CST, #4021T	Rb	270	1:1000
Vinculin	Sigma, #V9131	Ms	116	1:500

[1] Ms: mouse; Rb: rabbit.
